# Twisted Troubles: A Rare Case of Intestinal Obstruction Due to Endometriosis and a Review of the Literature

**DOI:** 10.3390/clinpract14050160

**Published:** 2024-09-27

**Authors:** Ionut Eduard Iordache, Luana Alexandrescu, Alina Doina Nicoara, Razvan Popescu, Nicoleta Leopa, Gabriela Baltatescu, Andreea Nelson Twakor, Ionut Tiberiu Tofolean, Liliana Steriu

**Affiliations:** 1 Department of General Surgery, “Sf. Apostol Andrei” Emergency County Hospital, 145 Tomis Blvd., 900591 Constanta, Romania; eduardiordache@yahoo.com (I.E.I.); razvanpop2000@yahoo.com (R.P.); gherghe_nicoleta02@yahoo.com (N.L.); lilianasteriu@yahoo.com (L.S.); 2 Faculty of Medicine and Pharmacy Constanta, Ovidius University, 900470 Constanta, Romania; alina.nicoara@365.univ-ovidius.ro (A.D.N.); tofoleanioan@yahoo.com (I.T.T.); 3 Gastroenterology Department, “Sf. Apostol Andrei” Emergency County Hospital, 145 Tomis Blvd., 900591 Constanta, Romania; 4 Internal Medicine Department, “Sf. Apostol Andrei” Emergency County Hospital, 145 Tomis Blvd., 900591 Constanta, Romania; andreea.purcaru@365.univ-ovidius.ro; 5 Clinical Service of Pathology, “Sf. Apostol Andrei” Emergency County Hospital, 145 Tomis Blvd., 900591 Constanta, Romania; gabrielabaltatescu@yahoo.com

**Keywords:** endometriosis, intestinal occlusion, volvulus, ileo-caecal anastomosis, extragenital

## Abstract

Background and Objectives: Intestinal endometriosis is an exceptionally rare cause of intestinal obstruction. This case report and literature review aim to highlight the clinical presentation, diagnostic challenges, and surgical management of this condition. Materials and methods: We report the case of a 50-year-old female patient who presented diffuse abdominal pain, nausea, vomiting, a distended abdomen, and an absence of intestinal transit for gas and faeces. Initial symptoms included flatulence and constipation, which gradually worsened for two months prior to the patient’s hospital admission, leading to acute intestinal obstruction. Diagnostic investigations, including blood tests, ultrasound (USG), X-ray, and a contrast-enhanced computer tomography (CT) scan, revealed significant small bowel dilatation and an ileal volvulus. The patient underwent urgent hydro-electrolytic and metabolic rebalancing followed by a median laparotomy surgical procedure. Intraoperative findings included a distended small intestine and an obstructive ileal volvulus, and required an 8 cm segmental enterectomy and terminal ileostomy. Results: Postoperative recovery was slow but favourable, with a gradual digestive tolerance. Histopathological examination of the resected ileum revealed intestinal endometriosis characterized by a fibro-conjunctive reaction and nonspecific chronic active inflammation. Five months later, the patient underwent a successful reversal of the ileostomy with a mechanical lateral anastomosis of the cecum and ileum, resulting in a favourable postoperative course. Conclusions: This case underscores the importance of considering intestinal endometriosis in women presenting with unexplained gastrointestinal symptoms and highlights the need for timely surgical intervention and careful postoperative management. Further research is required to better understand the pathophysiology and optimal treatment strategies for intestinal endometriosis.

## 1. Introduction

Endometriosis is characterized by the existence of endometrial glands and stroma-like lesions outside of the uterus. These lesions can be seen on many organs throughout the body. This condition triggers a persistent inflammatory response [1].

While some women with endometriosis experience painful symptoms or infertility, others have no symptoms at all. Despite being a benign disorder, endometriosis significantly impairs the fertility of the affected women [2].

The precise prevalence of endometriosis is uncertain; however, it is estimated to affect up to 15% of women in their reproductive years and around 70% of women experiencing chronic pelvic pain and/or infertility [3,4].

Although the exact cause of endometriosis is not known, there are multiple theories about the development of these lesions. Retrograde menstruation is a potential mechanism [5]. Sampson’s theory suggests that during retrograde menstruation through the Fallopian tubes, some of the menstrual debris attaches to peritoneal surfaces and invades the tissue [6].

Implantation of endometrial tissues into the peritoneal wall and other extragenital locations, as well as subsequent lesion development and the recruitment of other cell types promoting invasion and proliferation, leaves many unanswered questions about the basic mechanisms involved here [7].

The recent EndoCost Study conducted by the World Endometriosis Research Foundation (WERF) has revealed that the expenses incurred from treating women with endometriosis in specialized centres are significant. This creates an economic burden that is at least equivalent to the burden caused by other chronic illnesses such as diabetes mellitus, Crohn’s disease, and rheumatoid arthritis [8].

In addition to the financial burden, endometriosis has a substantial impact on other aspects of women’s lives, such as their social and sexual relationships, as well as their work and education [9,10,11]. 

The objective of this case report and literature review is to highlight the diagnostic challenges and surgical management of a rare case of intestinal obstruction caused by extragenital endometriosis. By presenting this case and reviewing relevant literature, we aim to add to the understanding of the clinical presentation, appropriate diagnostic approaches, and effective treatment strategies for healthcare professionals encountering similar cases.

## 2. Materials and Methods

A literature review was also conducted to gather relevant studies on the occurrence of intestinal obstruction due to endometriosis. The primary objective of this review was to assess the clinical presentation, diagnostic methods, treatment options, and outcomes associated with this rare condition. We performed a search on the PubMed database using the keywords “intestinal endometriosis bowel obstruction”. The filters applied were: Free full text, Case Reports, English, Adult: 19+ years, and January 2020–July 2024. 

The exclusion criteria were studies focusing on endometriosis without gastrointestinal involvement, articles lacking sufficient clinical detail or those that were not peer-reviewed.

**Figure 1:**
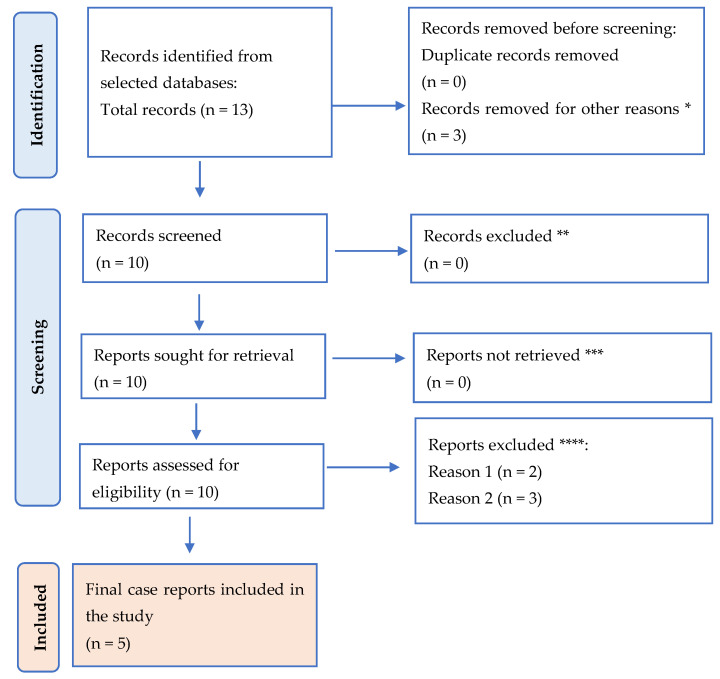
PRISMA flow diagram for the results. * studies are not relevant for the present review. ** studies do not help us to provide an answer to the current research. *** unable to find the full text of the study. **** Reason 1—study on animals/Reason 2—wrong setting/Reason 3—research question not relevant.

This review was outlined here using the Preferred Reporting Items for Systematic Review and Meta-Analysis (PRISMA) guidelines (Figure 1). The search resulted in 13 titles. 

This study was conducted in accordance with the principles outlined in the Declaration of Helsinki. The research protocol was approved by the Ethics Committee of “Sf. Apostol Andrei” Emergency County Hospital, Constanta, Romania (protocol code 267/11.10.2023). Informed consent was obtained from the patient involved in this case report prior to any procedures, ensuring her understanding and voluntary participation. Confidentiality and privacy were maintained throughout the study, and no identifying information has been disclosed. The authors declare no conflicts of interest.

## 3. Case Report

Patient Information: This case report details the medical intervention for a 50-year-old nulliparous female patient who was admitted to the Surgical Clinic at Constanta County Hospital in Romania. The patient presented with widespread abdominal pain, accompanied by nausea, vomiting, a swollen abdomen, and an absence of intestinal transit for gas and faeces. The symptomatology started 14 days prior to the emergency intervention, and the patient was in the 18th day of her menstrual cycle at the time of admission. The patient had a body mass index (BMI) of 20.5 kg/m^2^, with a weight of 62 kg and a height of 174 cm. The patient had no previous gynaecological evaluation or personal or family medical history relevant to her current condition.

Clinical Findings: The patient reported that her symptoms began approximately 2 months prior to hospital admission. Initially, she experienced excessive gas and constipation, which progressively worsened. The severity of these symptoms escalated, leading to a complete intestinal blockage 4 days before she sought emergency medical care.

On physical examination, the patient appeared in moderate distress. Her abdomen was visibly distended, with diffuse tenderness on palpation. There was no evidence of bowel sounds, and a rectal examination revealed an empty rectum. The patient’s vital signs were stable, but she reported increasing pain and discomfort.

### 3.1. Timeline

Two months prior to admission: onset of excessive gas and constipation.

Four days prior to admission: worsening of symptoms, leading to a complete intestinal blockage.

Day of admission: emergency admission to the Surgical Clinic due to severe abdominal pain, vomiting, and lack of intestinal transit 4 h after arriving at the hospital.

### 3.2. Diagnostic Assessment

The initial diagnostic workup included blood tests, an ultrasound, and a contrast-enhanced CT scan of the abdomen and pelvis. Blood tests were unremarkable except for mild leucocytosis.

The differential diagnosis initially included internal hernia, volvulus, and other causes of acute bowel obstruction. It also included an ileo-caecal tumor, Crohn’s disease with ileal localization and stenosis, an advanced right adnexal tumor, a retroperitoneal tumor with invasion in the caecal area, and internal hernia in the para-caecal fossae.

The diagnostic findings, combined with the patient’s history and clinical presentation, strongly supported the diagnosis of a volvulus.

### 3.3. Imagistic Findings

Comprehensive imagistic investigations were performed, including ultrasounds, X-rays, and contrast-enhanced CT scans to achieve an accurate diagnosis and detailed assessment of the patient’s condition. 

**Figure 2:**
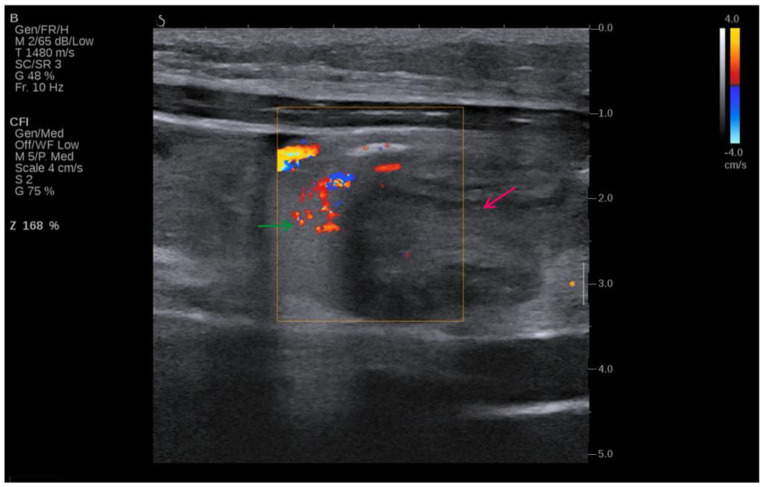
Ultrasound image of the volvulus—intestine twists around itself and its mesentery (red arrow), “whirlpool sign” (green arrow).

Figure 2 shows that initial imaging with ultrasound was used due to its non-invasive nature and ability to provide real-time imaging, allowing for the evaluation of soft tissue structures and the detection of fluid collections. 

This ultrasound image of the patient shown in Figure 2 indicates volvulus, and it can be seen where the intestine twists around itself and its mesentery (red arrow). The image is enhanced with Doppler colour flow mapping, showing swirling blood flow patterns, which is characteristic of the “whirlpool sign” (green arrow). The Doppler settings, as noted on the left side of the image, indicate adjustments made for optimal visualization of the blood flow, capturing the dynamic nature of the twisting vessels. The colour flow imaging reveals areas of both high and low velocities, portrayed in shades of red and blue, corresponding to blood flow towards and away from the transducer. This sign is critical in diagnosing volvulus, as it directly visualizes the torsion of the bowel and its mesenteric vessels, proving sufficient information for an emergency surgical procedure. The surrounding tissue appears hypoechoic, consistent with fluid accumulation due to obstruction. 

**Figure 3:**
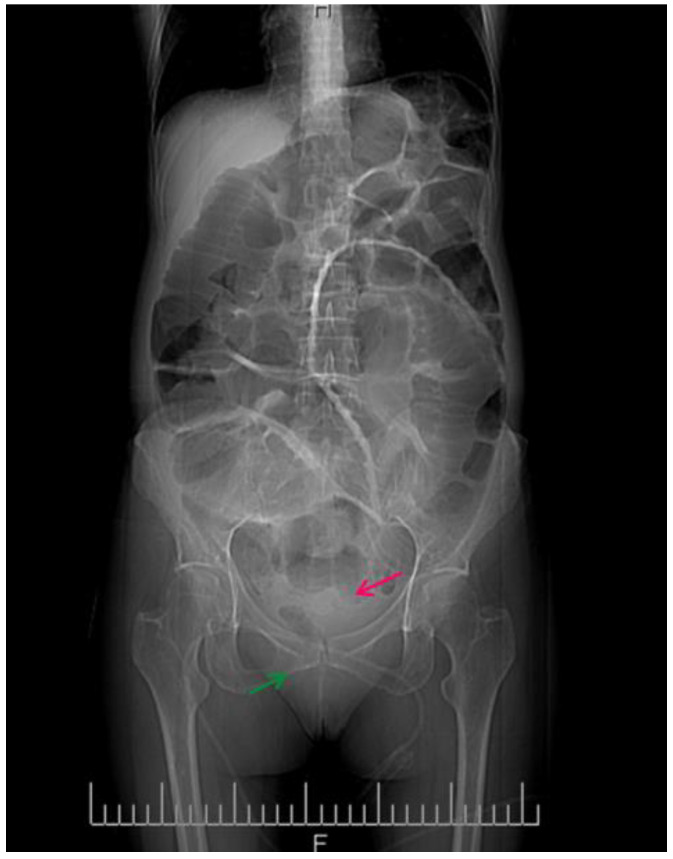
CT image of the intestinal volvulus—“whirlpool sign” (red arrow), air loops (green arrow).

Figure 3 shows the contrast-enhanced CT scan of the abdomen and pelvis being performed, which provided a detailed view of the abdominal cavity.

This abdominal X-ray shows multiple dilated loops of the small bowel with prominent air–fluid levels, a classic radiographic feature indicative of bowel obstruction. The image reveals significant distension of the bowel loops, which are visibly outlined by the accumulated air and fluid, highlighting the extent of the obstruction. The presence of these air–fluid levels is a critical diagnostic sign, suggesting that the bowel segments are filled with both air and liquid, which occurs when there is a blockage preventing the normal passage of intestinal contents. The upright position of the X-ray allows for a clear visualization of these levels, helping in the confirmation of the diagnosis. 

**Figure 4:**
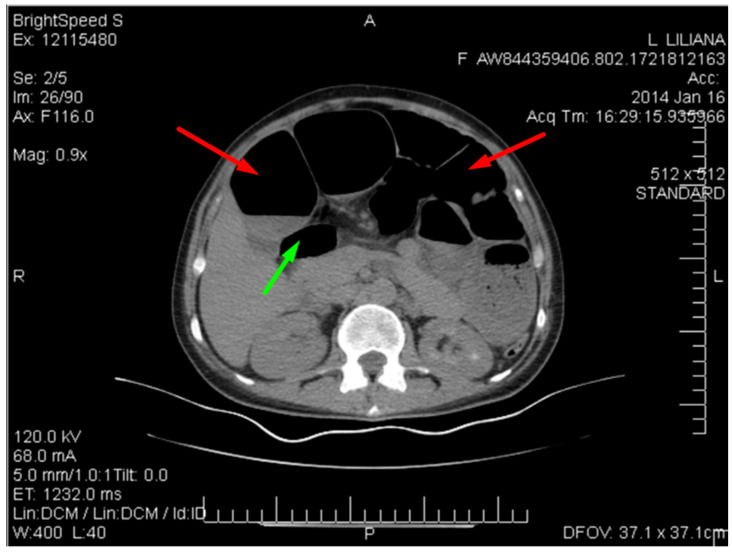
CT image of multiple dilated loops of small bowel with air (red arrows), and fluid (green arrow).

Figure 4 below shows multiple dilated loops of the small bowel with air.

This distension is seen in many cases of small bowel obstruction, leading to the buildup of gas and fluid in the proximal segments. The absence of air in the distal bowel segments further supports the diagnosis of obstruction.

**Figure 5:**
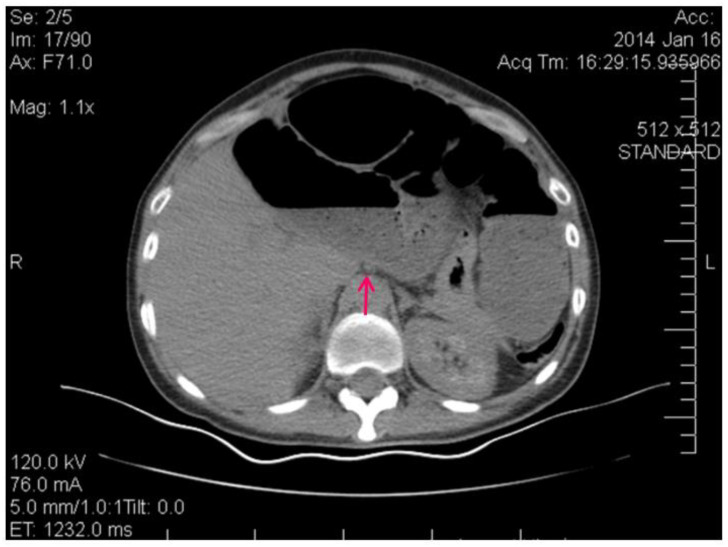
CT image of the “Whirlpool sign”— spiralling appearance of the mesenteric vessels (red arrow) suggestive of a small bowel volvulus.

Figure 5 shows the spiralling appearance of the mesenteric vessels.

The CT scan revealed distention of the small intestine and detected an ileal loop that appeared looped around a blood vessel, validating the diagnosis of an intestinal volvulus. 

The USG findings, in conjunction with the X-ray and contrast-enhanced CT, were crucial in detecting the intestinal obstruction produced by the volvulus and provided guidance for the eventual surgical operation. The integration of these visual tools allowed for a thorough evaluation, facilitating prompt and efficient handling of this intricate situation.

### 3.4. Intraoperative Findings

**Figure 6:**
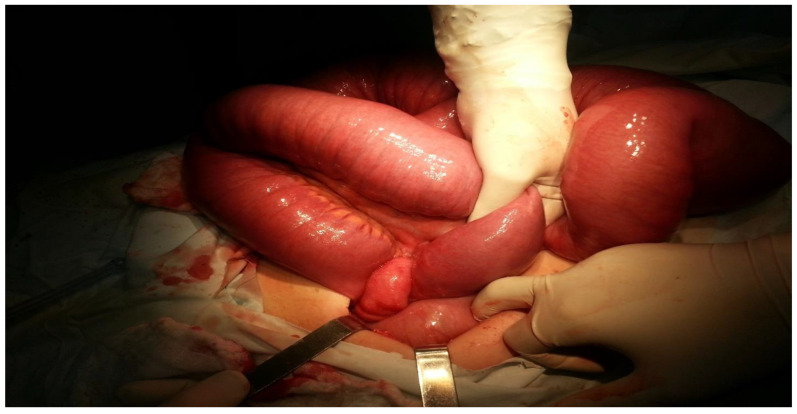
Volvulus of the small intestine.

**Figure 7:**
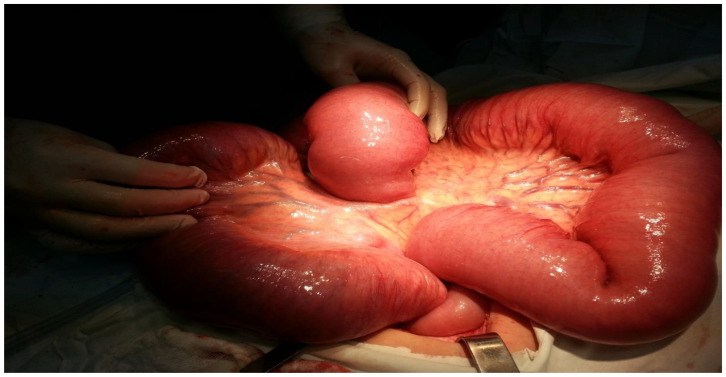
The degree of distension of the small intestine.

A median laparotomy was performed following hydro-electrolytic and metabolic rebalancing. During the procedure, it was observed that the entire intestinal wall was enlarged, with the small intestine measuring approximately 8 cm in diameter (see Figure 6 and Figure 7).

Figure 6 describes the small intestine being severely distended with visible signs of intestinal volvulus, with the ileum being twisted around its vascular axis. The surgical intervention involved the careful manipulation and dissection of the twisted loop to relieve the obstruction, addressing the patient’s acute intestinal blockage.

Figure 7 displays a markedly enlarged portion of the small intestine, illustrating the severity of the obstruction. The surgical team inspected the affected area, which was significantly congested, suggesting a lengthy obstruction and buildup of intestinal contents. The distension is evident from the smooth, taut appearance of the intestinal walls. This emphasizes the severity of the obstruction and the critical need for surgical intervention to relieve the blockage and restore normal intestinal function.

**Figure 8:**
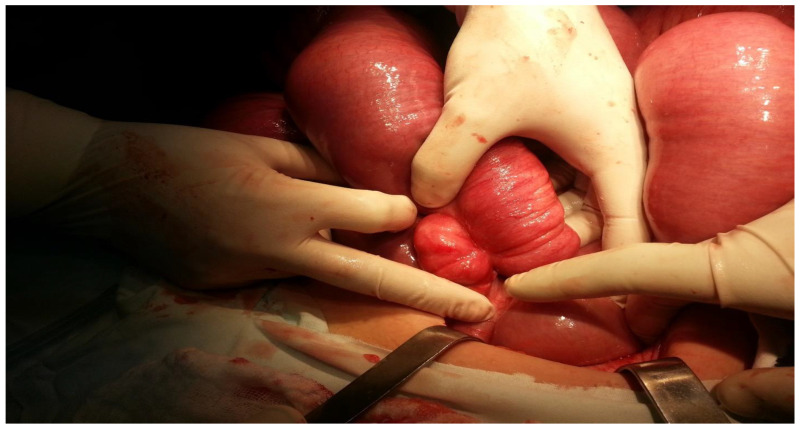
The appearance of the ileal loop before devolution.

**Figure 9:**
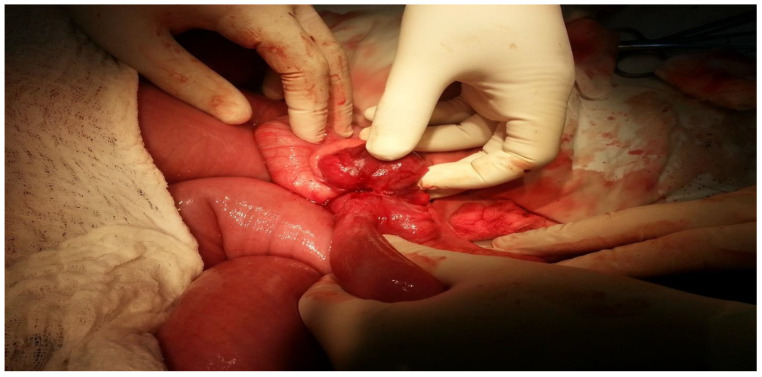
The appearance of the ileal loop after devolution.

Further, the obstruction was located at the terminal ileum to the right of the cecum, caused by an ileal volvulus, leading to almost complete obstruction of the intestinal lumen (Figure 8 and Figure 9).

This intraoperative image illustrates the appearance of the ileal loop before devolution. The surgeon handled the twisted segment of the ileum. As Figure 8 shows, the bowel loop is tightly twisted around itself. The surrounding intestinal tissue is swollen and congested, indicative of impaired blood flow and increased pressure within the loop. 

Figure 9 shows the ileal loop after successful devolution. The previously twisted segment has been carefully untwisted, revealing the underlying bowel tissue. Despite the relief of the volvulus, the affected segment shows significant signs of trauma and inflammation, with marked areas of redness and swelling. The surrounding intestinal loops appear less distended compared to the pre-devolution state, indicating some reduction in intraluminal pressure. The surgical procedure, which involved meticulous blunt dissection to untwist the ileum, was fundamental in restoring bowel continuity and preventing further damage due to ischemia. 

### 3.5. Surgical Intervention

The critical steps in managing the ileal volvulus, from identifying and untwisting the obstructed segment to assessing the viability of the affected bowel, led to a decision for limited enterectomy and the creation of a terminal ileostomy to ensure patient recovery and to prevent recurrence.

**Figure 10:**
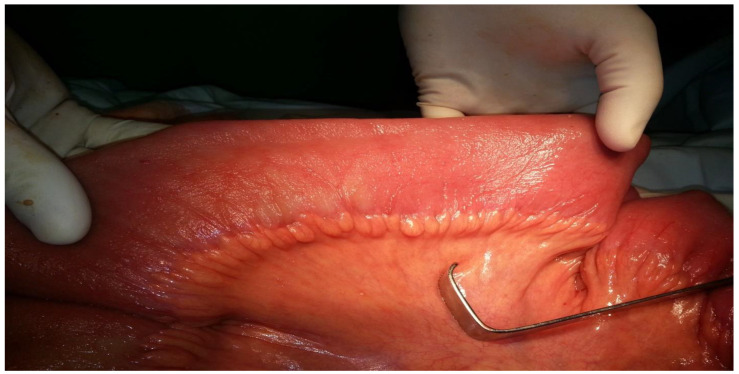
The small intestine after decompression.

**Figure 11:**
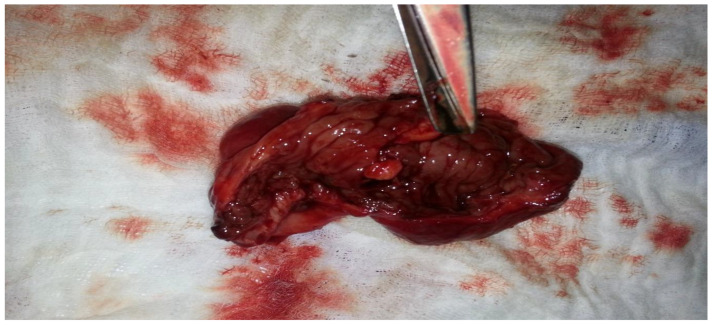
Enterectomy piece, with evidence of tumor injury.

Through blunt dissection, the ileum was decompressed. However, due to the presence of lesions over a 5–6 cm segment, an 8 cm limited enterectomy was performed. Anterograde decompression of the intestinal frame was achieved, and a terminal ileostomy was constructed in the right iliac fossa (Figure 10 and Figure 11).

The tumor was located precisely at the level of the ileocecal valve, where it caused a progressive stenosis, leading to the distension of the small intestine in an attempt to overcome the obstruction. However, the intestine was excessively distended, which contraindicated the creation of an anastomosis. Therefore, a minor enterectomy of approximately 8 cm starting from the ileocecal valve was performed, followed by a terminal ileostomy.

This intraoperative image shows how the small intestine looks after successful decompression. The bowel wall, now relieved of the intense distension caused by this obstruction, appears more relaxed and less enlarged. The surgical team managed to decompress the bowel, reducing the intraluminal pressure and improving the acute distension that was evident preoperatively. The small intestine’s mucosal folds are visible, and the tissue looks viable, indicating that the blood supply has been preserved. This stage of the procedure is crucial in ensuring that the bowel returns to a more normal state and function, preventing further complications, such as ischemia or perforation. The decompression process is an essential step following the correction of a volvulus, allowing for a more thorough assessment of the bowel’s condition and guiding the surgical team in deciding the subsequent steps, which in this case included an enterectomy and creation of a terminal ileostomy.

Figure 11 shows the resected segment of the ileum following the enterectomy. The excised tissue, marked by significant pathological changes, was removed due to the presence of severe lesions and evidence of a tumor injury, which contributed to the obstruction and volvulus of the small intestine. The estimated blood loss was less than 100 mL. The resection was performed to remove the damaged portion of the bowel, ensuring that only healthy and viable tissue remained. The resected piece displays the extent of the damage, with visible signs of chronic inflammation and structural distortion caused by the underlying endometriosis, which was later proved through the histopathological analysis. This crucial step in the surgical procedure not only relieves the immediate obstruction but also helps prevent recurrence and further complications. 

### 3.6. Histopathological Findings

According to the AAGL 2021 classification, endometriosis is categorized into four grades based on the extent and severity of the disease: minimal (Grade I), mild (Grade II), moderate (Grade III), and severe (Grade IV) [12]. This classification considers the distribution, depth, and severity of endometriotic lesions, including superficial implants, DIE, and associated adhesions [13]. The present case, involving a 50-year-old female patient with a diagnosis of intestinal obstruction due to endometriosis, corresponds to Grade II (mild). This classification is based on the findings of mild adhesions and the presence of endometriotic lesions that were primarily localized, without extensive infiltration or severe distortion of the pelvic anatomy.

**Figure 12:**
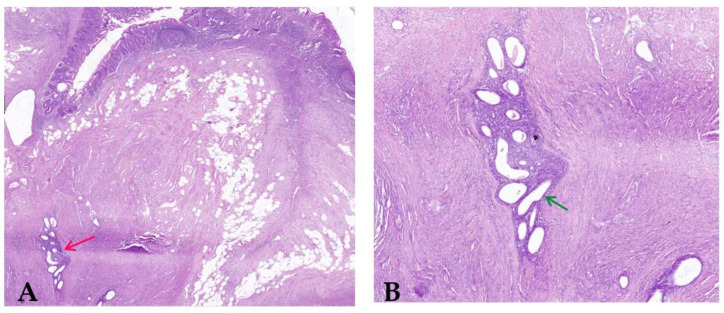
(**A**) Parietal endometriosis (red arrow) of the intestinal wall with normal intestinal mucosa (H&E stain; original magnification 40×); (**B**) Clusters of endometrial glands and stroma (green arrow) into the muscularis propria of the intestinal wall with discrete inflammation (H&E; original magnification 200×).

The recognition of the disease as Grade II endometriosis is significant as it correlates with the clinical presentation and the surgical findings. Although the patient presented with significant gastrointestinal symptoms leading to an intestinal obstruction, the overall distribution of the endometriotic lesions was limited, which aligns with the mild grade classification. Histopathological analysis of the resected ileum revealed the unexpected presence of intestinal endometriosis, a rare and intriguing finding. In this case report, endometriosis was detected in the intestinal tract, which is not generally where endometrial tissue is seen. This investigation revealed the presence of endometrial glands and stroma within the wall and a consequent fibro-conjunctive reaction. As can be seen in Figure 12, this response is recognized because of the fibrous and connective tissues that develop locally due to persistent irritation and inflammation induced by the presence of extra-genital endometrial tissue.

In addition, the histopathological exam revealed the presence of nonspecific chronic active inflammation due to structural changes in the intestinal tissue, but also signs of an ongoing immune response which contributed to the patient’s symptoms. The discovery of intestinal endometriosis in this patient highlights the need for considering this diagnosis in women presenting with unexplained gastrointestinal symptoms, especially when conventional diagnostic tests do not reveal typical causes of bowel obstruction.

### 3.7. Postoperative Course

The patient’s post-op course was characterized by a slow yet steady recovery, marked by a gradual resumption of digestive tolerance. She was administered antibiotic therapy with third generation Cephalosporin. 

This evolution is consistent with typical outcomes following major abdominal surgeries, where the initial days post-operation often involves managing pain, preventing infection, and ensuring the return of gastrointestinal function [14]. The patient’s gradual improvement in digestive tolerance was a positive indicator of intestinal recovery, often observed through the reintroduction of oral food intake and the progression from liquids to solid foods. Significant improvement in both general and mental health is also noteworthy, reflecting the alleviation of chronic symptoms and the psychological relief from resolving a distressing and painful condition [15]. Such recoveries, while slow, are crucial milestones in postoperative care, as they indicate the body’s successful adaptation and healing process following surgical intervention. This improvement aligns with literature suggesting that thorough postoperative management, including nutritional support and mental health care, plays a vital role in the overall recovery of patients after complex surgeries like those involving bowel resection and ileostomy formation [16,17,18].

### 3.8. Follow-Up Surgery

Five months later, the patient returned for a scheduled procedure to restore digestive continuity. This involved the reversal of the ileostomy, which was accomplished through a mechanical lateral anastomosis of the cecum and ileum using a 60 mm gastrointestinal anastomosis (GIA) stapler. This surgical technique aimed to re-establish the normal flow of intestinal contents and the procedure was meticulously performed to ensure a secure and functional anastomosis, thus reducing the risk of short- and long-term complications. The patient’s recovery was successful, with no reported complications. 

## 4. Discussion and Literature Review

Endometriosis is characterised by a broad spectrum of clinical signs and symptoms depending of which sites are affected [19]. Symptoms, though often diverse and puzzling, are usually the consequence of extra-genital endometrial tissue, which leads to fibrosis in the affected organ [20]. In general, pain and/or bleeding not related to the menstrual cycle could offer clues that we are in the presence of endometriosis. Other symptoms, however, can confuse medical teams [21,22]. When the endometrial cells are deeply infiltrated, the endometriotic lesions also affect the nervous system of the pelvic floor, causing chronic pain [23].

Two subtypes of endometriosis are differentiated by gross and microscopic inspection: superficial endometriotic implants and deeply infiltrating endometriosis (DIE) [24]. Superficial endometriotic implants are shallow, <5 mm in depth, and typically scattered throughout the abdominal–pelvic peritoneal surfaces [25,26]. Its characteristic histology is described as deposits of endometrial-like glands and stroma with evidence of menstrual shedding [6]. 

A second manifestation is the deeply infiltrating way in which the endometriotic lesion infiltrate >5 mm beneath the peritoneum [27]. These lesions show marked fibrosis and smooth muscle hyperplasia in addition to the classical combination of endometrial glands and stroma [28,29].

### 4.1. Extragenital Endometriosis 

Table 1 below provides a comprehensive overview of recent case reports and a retrospective study involving intestinal obstruction due to endometriosis, covering a range of clinical presentations, diagnostic methods, treatments, and outcomes. We conducted our research using the PubMed data base and we found that over the past 4 years, these five papers highlight the variability of symptoms, such as abdominal pain, vomiting, constipation, and chronic pelvic pain, that can lead to the identification of this rare condition. 

The diagnostic approaches typically involved advanced imaging techniques like CT scans, MRIs (magnetic resonance imaging), and diagnostic laparoscopies, which were crucial in confirming the presence of endometriotic lesions causing bowel obstruction. Surgical intervention was the primary treatment option, with procedures ranging from the laparoscopic resection of affected bowel segments to more extensive surgeries like segmental bowel resection and colectomy. 

Collectively, these case studies provide critical insights into the effective management of bowel obstruction caused by endometriosis, demonstrating the importance of individualized treatment plans and the need for ongoing research to optimize patient outcomes.

The prevalence of non-genital endometriosis in patients with pelvic endometriosis has been estimated to be up to 12% [35]. The main locations are the colon, urinary tract, abdominal cavity, lungs, skin, and the nervous system [15,22].

Symptoms depend on the site of the disease. Cyclicity of symptoms is usually present, at least in early stages, and may be the only clue that leads to the diagnosis of endometriosis. Diagnosis is usually made by histological confirmation, which is important to exclude other pathology, such as malignancy [36].

Treatment will also depend on the site. If complete excision is possible, this is the treatment of choice; when this is not possible, long-term medical treatment is necessary [13]. 

Jubanyik et al. [37] and Joseph et al. [38] showed in their research that the principles of medical treatment for pelvic endometriosis will similarly apply for extragenital endometriosis.

### 4.2. DIE of the Small Bowel, Large Bowel, and Appendix

DIE involving the bowel affects 10–12% of women diagnosed with endometriosis [39]. Mohr et al. [40] reported on a series of 187 women with endometriosis involving the bowels, and the most common symptoms were pelvic pain 99%, dyschezia 74%, back pain 55%, constipation 74%, diarrhoea 41%, melena 16%, and nausea 8%. 

In another comprehensive literature review, Musat et al. [41] estimated that 7% to 37% of patients with endometriosis in the small intestines. The authors identified that the most common sites of GI (gastrointestinal tract) endometriosis are the rectosigmoid (51%), the appendix (15%), small bowel (14%), the rectum (14%), and the caecum and colon (5%) [42].

The surgical treatment dilemma for endometriosis of the small bowel, similar to management of the colon and rectum, is whether to shave the lesion from the surface of the bowel, remove a disc of bowel wall around the endometriosis, or resect a segment of bowel. 

Donnez et al. [43] compared 500 women undergoing different surgical procedures for the removal of DIE. Their results showed that complete pain relief in the immediate postoperative period was significantly more likely with partial bowel resection than with shaving only, 92% versus 80% [43]. 

### 4.3. Ultrasound Findings of Intestinal Volvulus Due to Extragenital Endometriosis

Typical ultrasound features of intestinal volvulus include the “whirlpool sign”, which represents the twisted bowel loops and the mesentery around the point of torsion. This feature was first described by Vijayaraghavan in 2004 [44], who noted that this is a critical ultrasound finding representing the torsion of anatomical structures, such as the intestines or the adnexa, around their supporting vasculature. Higashide et al. [45] also added that this sign is characterized by the presence of a spiral or whirlpool-like appearance of the twisted bowel loops and the mesentery, or the twisted adnexa, visible on Doppler ultrasound as swirling of the vessels [45].

Rousslang et al. [46] has noted that there might be reduced or absent peristalsis in the affected segment, and increased echogenicity can be seen in the mesentery due to congestion and edema. The presence of free fluid in the abdominal cavity can also be noted, which indicates a more advanced or complicated volvulus [46]. Barra et al. [47] have identified that in the context of endometriosis, these ultrasound findings can be further complicated by the presence of endometriotic lesions, which might appear as hypoechoic or heterogeneous masses within the bowel wall or attached to the surrounding structures. Other studies have also shown that transvaginal and transabdominal ultrasounds can help in identifying these lesions, especially when combined with a detailed patient history and clinical examination. For instance, Hudelist et al. [48] have highlighted the significance of transvaginal ultrasonography in the diagnosis of deep infiltrating endometriosis that affects the colon. In such cases, the lesions are typically observed as irregular nodules or a thickening of the gut wall. 

Bazot et al. [49] have conducted research into the use of transvaginal sonography in diagnosing intestinal endometriosis. Their results indicated that transvaginal ultrasound had a sensitivity of 91% and a specificity of 98% in accurately detecting rectosigmoid endometriosis when compared to the findings from surgery. Similarly, Guerriero et al. [50] performed a comprehensive evaluation and statistical analysis that verified the diagnostic accuracy of transvaginal ultrasonography in detecting intestinal endometriosis. They also reported combined sensitivity and specificity values of 91% and 98%, respectively. 

The combination of these findings—the whirlpool sign, dilated bowel loops, and the presence of endometriotic lesions—can provide strong indicators for diagnosing intestinal volvulus due to endometriosis. However, further imaging with CT or MRI is often required for a more comprehensive assessment and to plan the appropriate surgical intervention.

## 5. Conclusions

This case report highlights the significance of considering intestinal endometriosis as a differential diagnosis in women presenting with unexplained gastrointestinal symptoms. The identification of intestinal endometriosis as the cause of the patient’s bowel obstruction highlights the diagnostic challenges and the critical need for a suspicion, especially in patients with known endometriosis or symptoms suggestive of this condition. 

In this case report, histopathological examination played a crucial role in confirming the diagnosis of intestinal endometriosis, revealing endometrial glands and stroma within the bowel wall and associated fibro-conjunctive reaction. This case adds to the limited but growing body of literature on extragenital manifestations of endometriosis, particularly involving the intestines. It also highlights the necessity for comprehensive postoperative care to ensure a smooth recovery and the potential for restoring normal gastrointestinal function. Further research is needed to better understand the pathophysiology of intestinal endometriosis and to develop optimal diagnostic and treatment strategies for this rare but potentially lethal condition.

## Figures and Tables

**Table 1:** Literature review of five articles included in the PRISMA flowchart covering bowel obstruction due to endometriosis.

Author	Year	Patient Details	Symptoms	Diagnostic Methods	Treatment	Outcome
Kondo et al. [30]	2020	Case series of 5 patients, aged 49, 48, 50, 37 and 43	Abdominal pain, bloating, bowel obstruction	Various tests: fecal occult blood test, colonoscopy, barium enema, CT scan, MRI	Laparoscopic sigmoidectomy, low anterior laparoscopic rectum resection, partial small bowel resection, high anterior laparoscopic colonic resection, and laparoscopic sigmoidectomy with side-to-side anastomosis	Good recovery for all 5 patients
Zepeda et al. [31]	2021	Case report, patient age 41	Abdominal pain, vomiting, constipation, dysuria and menorrhagia	CT scan, laparoscopy	Exploratory laparotomy at first, a large portion of distal ileum was resected along with the appendix	Complete recovery, symptom-free
Leborne et al. [32]	2022	Retrospective study, 165 women with DIE, median age 34	Dysmenorrhea, heavy menstrual bleeding, chronic pelvic pain, bowel obstruction	MRI, colonoscopy, histopathology	Hysterectomy was conducted in 22% of the cases, bowel segment or discoid resection surgery for almost 27%, and 77%—bowel shaving. 13.63% underwent a temporary protective ileostomy after their resection anastomosis procedure; 36.81% underwent ureterolysis, and 3.03% undertook vesical resection.	34 women had recurrence of the pain after a median duration of 12 months post-surgery; 23 women needed a second surgery after a median period of 22 months. According to the Clavien Dindo classification, 3 patients had grade IIIB complications and one grade IVA (intravenous anesthesia).
Ragab et al. [33]	2023	Case report, patient age 40	Abdominal pain, diarrhoea, vomiting, unable to tolerate oral intake, urinary tract infection.	Abdominal X-ray, CT scan, colonoscopic stenting was attempted, but the guide wire was unable to pass through the lumen, exploratory laparotomy.	Appendectomy stump, and extended left hemicolectomy was conducted due to compromised blood supply of the marginal arteries	Full recovery with no complications, oral food intake from the 1st day post-surgery, discharged on the 8th day post-op.
Thirumurthy et al. [34]	2024	Case report, patient age 35	2 years of abdominal pain, nausea and vomiting, no association with menstrual cycle	CT scan	Laparoscopic ileum resection and a limited resection of the ascending colon	Symptom-free at follow-up

Footnote: CT (Computed tomography), MRI (Magnetic resonance imaging), IVA (Intravenous anaesthesia), and DIE (Deeply infiltrating endometriosis).

## Data Availability

The data presented in this study are available on request from the corresponding author due to legal and ethical reasons.
